# Minimally invasive versus open surgery for acute achilles tendon rupture: an umbrella review of systematic reviews and meta-analyses

**DOI:** 10.3389/fsurg.2025.1671249

**Published:** 2025-10-28

**Authors:** Abdulmalik B. Albaker, Abdullah H. Alshahrani, Ismail H. AlMogbil

**Affiliations:** 1 Department of Orthopedics, College of Medicine, Majmaah University, Majmaah, Saudi Arabia; 2 Department of Orthopedic Surgery, College of Medicine, Qassim University, Buraydah, Saudi Arabia

**Keywords:** achilles tendon rupture, minimally invasive surgery, open surgery, systematic review, meta-analysis, surgical outcomes

## Abstract

**Background:**

Achilles tendon rupture is a common injury requiring surgical intervention. The choice between Minimally Invasive Surgery (MIS) and Open Surgery (OS) has been widely debated. This umbrella review synthesized the results of systematic reviews and meta-analyses comparing outcomes of MIS and OS for acute Achilles tendon rupture.

**Methods:**

A comprehensive search was conducted using PubMed, Embase, Cochrane Library, Web of Science, and Scopus. The AMSTAR-2 checklist was employed to assess the quality of the included systematic reviews and meta-analyses. Data on complication rates, surgical times, functional outcomes, and other patient-centric metrics were extracted and analyzed.

**Results:**

An aggregate of 6,480 participants were drawn from 7 included studies (not de-duplicated across overlapping trials). The primary outcomes were re-rupture and validated functional recovery scores; key complications included infection and sural nerve injury; secondary endpoints included operative time and return-to-sport. Searches, selection, and extraction followed prespecified criteria. Because the unit of analysis was published evidence syntheses and the underlying randomized trials overlapped with heterogeneous outcome definitions, results were synthesized qualitatively rather than pooled quantitatively. Across reviews, minimally invasive and open repair showed broadly comparable clinical effectiveness, with differences contingent on technique, perioperative protocols, and follow-up windows.

**Conclusion:**

MIS appears to offer significant advantages over OS for the repair of acute Achilles tendon ruptures, including reduced complication rates and faster recovery times, without compromising the effectiveness of the repair in preventing re-ruptures. However, the potential for nerve injury with MIS warrants careful consideration. Decisions regarding surgical techniques should be tailored to individual patient circumstances and the specific expertise of the surgical team.

## Introduction

The largest and strongest tendon in the human body, the Achilles tendon is essential to the locomotor movements of walking, running, and jumping (1). Its rupture is a severe handicap that is particularly common in athletes and middle-aged people who participate in irregular sports. Because of the potential effects on a patient's mobility, quality of life, and ability to resume sports or other activities, the management of acute Achilles tendon ruptures (ATRs) is still a topic of great clinical interest (2). Due to improvements in surgical methods and inconsistent results documented in the literature, there has been a continuous discussion concerning the best surgical strategy: open surgery vs. minimally invasive surgery (MIS) (3, 4).

The gold standard in tendon repair, open surgery, enables accurate tendon end apposition and direct visualisation. Due to the deeper incision needed, this approach may lower the risk of re-rupture but is linked to greater rates of wound complications, including infection and skin necrosis (5). However, MIS methods, which need fewer incisions and frequently use specialised equipment, are said to have a number of benefits (6–8). These include shorter recovery and operation times, fewer wound healing issues, and perhaps a quicker return to function. However, questions remain regarding the suitability of tendon approximation, the learning curve involved in using these methods, and the possibility of sural nerve injury (9).

The literature is full of investigations that seek to distinguish between the relative advantages and disadvantages of each surgical technique in the context of these opposing approaches (10–15). Different study designs, sample sizes, follow-up periods, and definitions of clinical goals such as re-rupture rates, functional outcomes, and complication rates can all have an impact on the various conclusions that these studies frequently present. A wide range of results might make it difficult for clinicians and patients to make decisions and obfuscate clear treatment guidelines.

Therefore, the purpose of this umbrella review is to synthesize and critically evaluate the findings of existing systematic reviews and meta-analyses comparing MIS and open surgery for acute ATR and provide a comprehensive appraisal of the evidence by identify gaps in current knowledge.

## Materials and methods

### Eligibility criteria

This umbrella review was carried out in accordance with the Preferred Reporting Items for Systematic Reviews and Meta-Analyses (PRISMA) criteria (16). Both a qualitative and quantitative summary of the findings was done when needed. The PECO (Population, Exposure, Comparator, Outcome) paradigm was applied in order to formulate the research question and direct the selection of the studies. Patients with acute ruptures of the Achilles tendon made up the population of interest. Treatments involving minimally invasive surgery were taken into consideration. The conventional open surgical techniques served as the comparative. Re-rupture rates, functional outcomes (as determined by instruments such as the Achilles tendon Total Rupture Score), and complication rates (which encompassed surgical site infections and nerve injury) were among the outcomes of interest. The different types of inclusion and exclusion criteria employed for this review have been elucidated through Table 1.

**Table 1:** Different inclusion and exclusion criteria established for this review.

Criteria	Inclusion	Exclusion
Study design	Only systematic reviews and meta-analyses were included.	Narrative reviews, case reports, and original research studies were excluded.
Participants	Studies involving patients with acute ATRs.	Studies involving patients with chronic Achilles issues, or non-rupture related interventions were excluded.
Interventions	Studies comparing minimally invasive surgery to open surgery.	Studies that did not directly compare these two surgical approaches were excluded.
Outcomes	Reviews that reported on at least one of the following outcomes: re-rupture rates, functional outcomes, or complication rates.	Studies lacking quantifiable outcome data on the specified measures were excluded.
Publication Status	Published and peer-reviewed articles were included.	Unpublished data, conference abstracts, and non-peer-reviewed articles were excluded.
Language	No limitation	
Date range		

### Database search protocol

The search was conducted using five major databases: PubMed, Embase, Cochrane Library, Web of Science, and Scopus. The search strategies were tailored to each database's specific indexing system and search capabilities, employing a combination of MeSH (Medical Subject Headings) terms, Emtree terms (for Embase), and free text terms, using Boolean operators to refine and combine search terms, the schematics of which have been shown through Table 2. Terminology was harmonized throughout the manuscript: “minimally invasive surgery (MIS)” and “open surgery (OS)” were used consistently, outcome labels were standardized (e.g., “re-operation”, “wound complications”, “return to activity”), and style edits improved clarity, spelling, and consistency in tables, figures, and text.

**Table 2:** Search strings and keywords utilised across the assessed databases.

Database	Search string
PubMed	((“Achilles Tendon”[MeSH] OR “achilles tendon rupture”[All Fields]) AND (“Surgical Procedures, Minimally Invasive”[MeSH] OR “minimally invasive surgery”[All Fields] OR “open surgical procedures”[MeSH] OR “open surgery”[All Fields]) AND (“meta-analysis”[Publication Type] OR “systematic review”[Publication Type]))
Embase	(“achilles tendon rupture”/exp OR achilles:ab,ti AND rupture:ab,ti) AND (“minimally invasive surgery”/exp OR “open surgical procedures”/exp OR minimally:ab,ti AND invasive:ab,ti OR open:ab,ti AND surgery:ab,ti) AND (“meta analysis”/exp OR “systematic review”/exp OR meta:ab,ti AND analysis:ab,ti OR systematic:ab,ti AND review:ab,ti)
Cochrane Library	[“Achilles Tendon”(MeSH) OR “achilles tendon rupture”] AND [“Surgical Procedures, Minimally Invasive”(MeSH) OR “minimally invasive surgery” OR “open surgical procedures” OR “open surgery”] AND (“Meta Analysis” OR “Systematic Review”)
Web of Science	[TS = (“Achilles Tendon” OR “achilles tendon rupture”) AND TS = (“minimally invasive surgery” OR “open surgery”) AND TS = (“Meta Analysis” OR “Systematic Review”)]
Scopus	[TITLE-ABS-KEY (achilles AND tendon AND rupture) AND TITLE-ABS-KEY (minimally AND invasive AND surgery OR open AND surgery) AND TITLE-ABS-KEY (meta-analysis OR systematic-review)]

### Variable extraction protocol

The data extraction process was conducted by two independent reviewers who used a standardized data extraction form to minimize bias and errors. Discrepancies between the reviewers were resolved through discussion or consultation with a third reviewer. The following data items were extracted from each included study:

1. **General information**: This included the author(s), year of publication, and country where the study was conducted. This information provided context about the geographical and temporal relevance of the findings.
2. **Study design**: Information about the methodology of the meta-analyses reviewed, including the number of studies included, total number of participants, and study design of the included primary studies (randomized controlled trials, cohort studies, etc.).
3. **Population characteristics**: Data were extracted on participant demographics such as age, sex, and baseline characteristics related to the severity of the Achilles tendon rupture and any comorbid conditions.
4. **Intervention details**: Specific details about the minimally invasive and open surgical techniques were recorded, including the type of procedure, surgical tools used, and any adjunct therapies employed post-surgery.
5. **Outcome measures**: The primary outcomes of interest were re-rupture rates, functional outcomes (measured by tools such as the Achilles tendon Total Rupture Score), and complication rates. Secondary outcomes might include operation time, hospital stay duration, and any measure of patient satisfaction or quality of life.
6. **Results**: Key findings related to the effectiveness and safety of minimally invasive vs. open surgery were extracted, including statistical measures such as risk ratios, mean differences, confidence intervals, and *p*-values.
7. **Conclusion and recommendations**: Summaries of the authors’ conclusions and any clinical recommendations were recorded to understand the implications of the meta-analyses’ findings.

No language restrictions were imposed. Non-English reports were translated by certified translators when available; when official translations were not obtainable, professional translation services were used and the output was checked by a bilingual clinician for technical accuracy and clinical terminology. Because multiple secondary reviews can include the same randomized trials, we mapped primary studies across all included reviews to identify overlap and mitigate double counting. When overlap was detected, we prioritized the most comprehensive and methodologically robust synthesis for quantitative summaries and treated overlapping evidence narratively to avoid inflating precision.

### Bias assessment protocol

The evaluation of bias was carried out utilising the AMSTAR-2 (A MeaSurement Tool to Assess systematic Reviews, version 2) checklist (17) in the included systematic reviews and meta-analyses. This tool was created with the express purpose of assessing the methodological quality of systematic reviews that comprise either non-randomized or randomised trials of healthcare interventions, or both.

## Results

**Figure 1:**
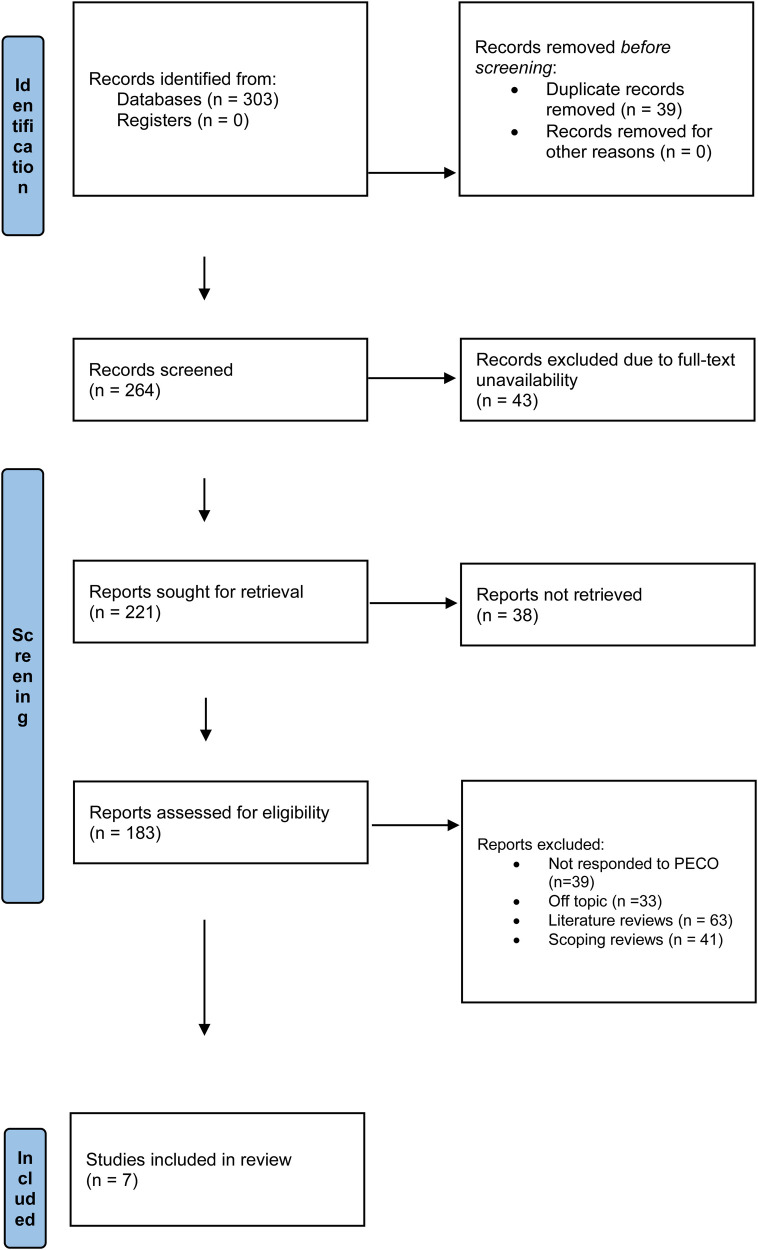
Article selection process representation for the review.

As seen in Figure 1, all 303 entries were found in all assessed databases in the first phase of the research selection process. There were 264 records remaining for evaluation after 39 duplicates were eliminated prior to screening. Since the full material could not be viewed at this time, 43 things were removed. As a result, an effort was made to acquire 221 reports. It was not possible to extract 38 of these records for additional analysis. Each of the 183 reports that were still available was carefully evaluated for eligibility. Out of the 39 studies that were judged to have violated the PICO requirements, 33 were considered off-topic, 63 were literature reviews, and 41 were scoping reviews. Seven systematic reviews and meta-analyses (18–24) were found to be appropriate for inclusion in the evaluation after these exclusion criteria were applied.

### AMSTAR tool observations

**Figure 2:**
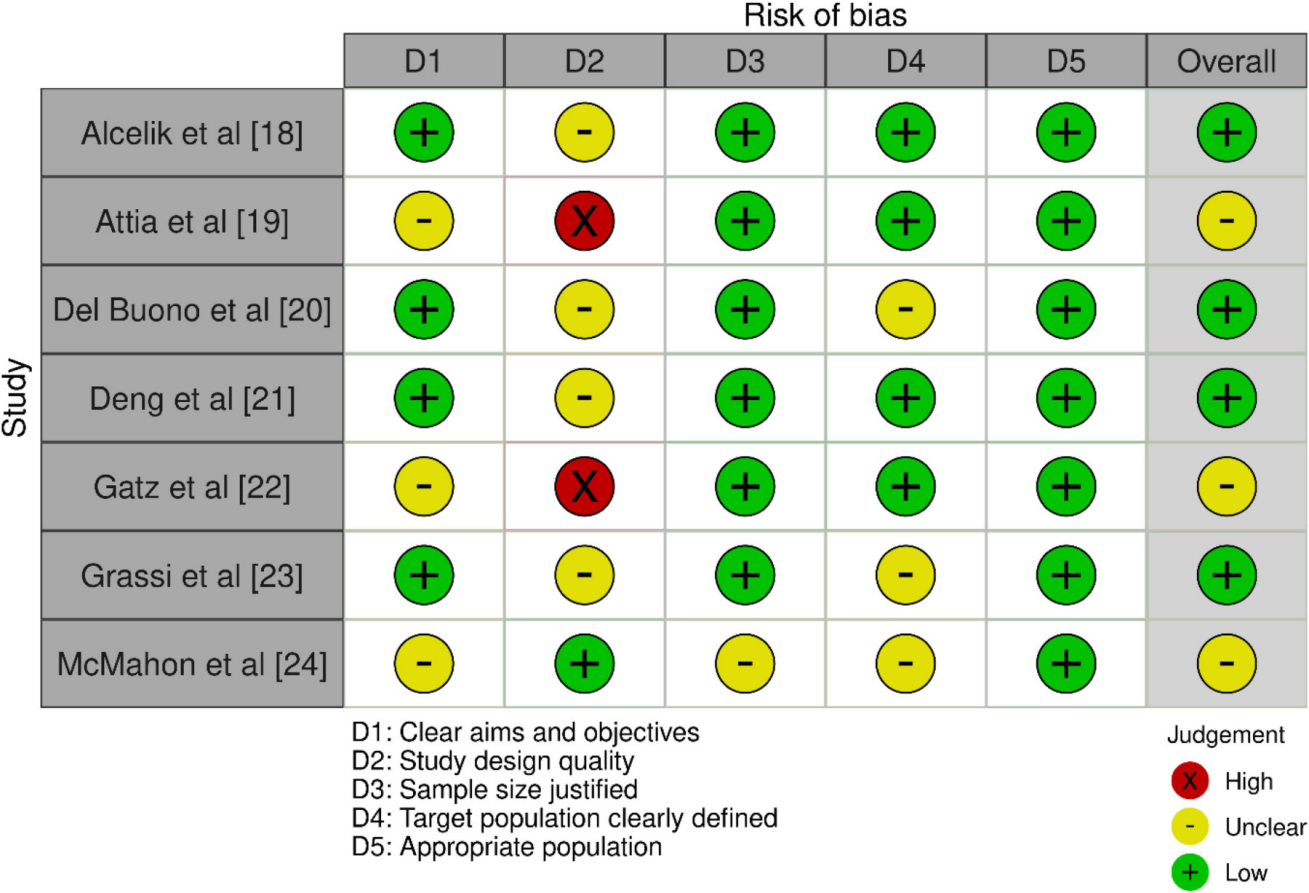
Bias assessment observations using AMSTAR-2 tool.

The AMSTAR-2 evaluation produced a range of findings (Figure 2). Alcelik et al. (18) showed a low risk of bias overall, although they did show a low risk of bias across some categories. Although the study design of Attia et al. (19) was of good quality, the study's aims and target populations were not well defined, which resulted in an unknown risk of bias overall. Despite having low risks in a number of categories, Del Buono et al. (20) and Grassi et al. (23) were both deducted for having imprecise study designs and target population definitions, which resulted in generally low risks of bias. Deng et al. (21) upheld a low risk in every domain, confirming an all-encompassing low risk of bias. However, while having a solid study design, Gatz et al. (22) had problems with poorly defined populations and ambiguous aims, which led to an unknown overall bias. These problems were shared by Attia et al. (19). McMahon et al. (24) encountered difficulties with imprecise evaluations in the majority of areas, such as study design and target population, resulting in an overall imprecise risk of bias.

### Demographic variables assessed

Table 3 shows the full picture of the demographic outcomes associated with different approaches for ATR as observed across the included investigations (18–24). Alcelik et al. (18) reviewed a total of 854 cases using a multimodal strategy that involved searching MEDLINE and EMBASE. Complications, re-rupture rates, sural nerve injury, and the patients' capacity to resume sports were the main areas of attention for their evaluation. Attia et al. (19) examined 522 cases from databases containing several languages while adhering to PRISMA principles. The assessment was broadened by their work to include sural nerve injury, skin problems, different rates of infection (both superficial and deep), functional scores like AOFAS/ATRS, surgery time, rates of re-rupture, adhesions, and ankle range of motion.

**Table 3:** Baseline characteristics of the included systematic reviews.

Study ID	Databases assessed	Total sample size (*n*)	Parameters assessed	Key outcomes assessed
Alcelik et al. (18)	MEDLINE, EMBASE, Current Controlled Trials, Center Watch, Trials Central, System for Information on Grey Literature in Europe, The UK National Research Register	854	Complications, re-rupture rates, sural nerve injury, return to sports	Post-operative complications, sural nerve injuries, re-ruptures, return to sports
Attia et al. (19)	PRISMA guidelines followed, databases unspecified but included literature in English, Spanish, Portuguese, and German	522	Sural nerve injury, skin complications, infection (deep/superficial), AOFAS/ATRS score, surgical time, re-rupture, adhesions, ankle range of motion, other complications	Sural nerve injury, skin complications, deep and superficial infection rates, re-rupture rate, surgical time, functional scores (AOFAS/ATRS), return to sport, ankle stiffness
Del Buono et al. (20)	Medline (PubMED), EMBASE, CINHAL, Cochrane, Sports Discus, Google Scholar	781	Complications, range of motion, cost, incision size, cosmetic outcome, patient satisfaction, pre-operative features, follow-up, surgical techniques, postoperative rehabilitation	Complications, range of motion, patient satisfaction, cosmetic outcomes, cost effectiveness
Deng et al. (21)	Medline, Embase, Cochrane Central Register of Controlled Trials	1,465	Rerupture rates, infection rates, deep vein thrombosis (DVT) rates, sural nerve injury rates	Rerupture rates, infection rates, DVT rates, sural nerve injury rates
Gatz et al. (22)	PubMed, Scopus, Google Scholar	2,223	Minimally invasive vs. open repair for acute Achilles tendon rupture	Palpable knots, sural nerve palsy, surgery duration, wound necrosis, scar adhesions, infections, re-rupture
Grassi et al. (23)	MEDLINE/PubMed, Cochrane Central, EBSCOhost, ClinicalTrials.gov	358	Minimally invasive vs. open repair for acute Achilles tendon rupture	Complication rates, wound infection, subjective outcomes, rerupture rates, sural nerve injury, return to activity, ankle motion
McMahon et al. (24)	MEDLINE, Embase, Cinahl, PubMed, Ahmed, Greynet, SIGLE, NTIS, British Library, Current Controlled Trials, Cochrane Central	277	Minimally invasive vs. open surgery for Achilles tendon rupture	Re-rupture rates, deep and superficial infections, tissue adhesions, sural nerve injury, deep vein thrombosis, subjective outcomes

Del Buono et al. (20) used reputable databases like Medline and Cochrane to analyse data from 781 instances. Numerous pre- and post-operative factors, complications, range of motion, cost, incision size, cosmetic results, and patient satisfaction were all evaluated in this study. Deng et al. (21) examined the frequencies of rupture, infection, deep vein thrombosis (DVT), and sural nerve injury using a sizable sample size of 1,465 cases from sources such as Medline and the Cochrane Central Register of Controlled Trials. Their results offer vital information about the dangers of surgery and the frequency of major side effects including infections and DVT.

Gatz et al. (22) evaluated minimally invasive vs. open repair methods specifically for acute Achilles tendon rupture based on an analysis of 2,223 cases from PubMed and Scopus. They looked at things like palpable knots, palsy of the sural nerve, length of surgery, scar adhesions, infections, and re-rupture rates. With sample sizes of 358 and 277, respectively, Grassi et al. (23) and McMahon et al. (24) investigated the pros and cons of minimally invasive vs. open surgery for Achilles tendon rupture. Grassi et al. (23) assessed rates of complications, wound infection, subjective results, rates of rupture, injury to the sural nerve, return to activity, and ankle motion. The study conducted by McMahon et al. (24) evaluated sural nerve injury, deep vein thrombosis, tissue adhesions, re-rupture rates, and subjective results.

### Outcomes pertaining to MIS vs. OS

Table 4 shows the different outcomes pertaining to MIS and OS as analysed across the included reviews (18–24). Alcelik et al. (18) found that the MIS group had a complication rate that was significantly lower than the OS group, with an OR of 0.27 and a *P*-value of less than 0.00001. This suggests that MIS offers a strong safety advantage over OS. The MIS group had a greater return to sports rate, although the difference was not statistically significant (OR 1.54, *P* = 0.08). There was no discernible difference in the rates of sural nerve injury and re-rupture (*P* = 0.48 and *P* = 0.43, respectively) between the two groups.

**Table 4:** Inferences pertaining to MIS vs. OS as observed across the included reviews.

Study ID	Outcomes favouring MIS	Outcomes with no significant difference or favoring open repair	Overall inference drawn
Alcelik et al. (18)	– Complication rate significantly lower in MIS group (OR 0.27, *P* < 0.00001). – Higher, but not statistically significant, return to sports rate in MIS group (OR 1.54, *P* = 0.08).	– Sural nerve injury and re-rupture rates similar between groups (*P* = 0.48 and *P* = 0.43, respectively)	MIS tends to have fewer complications and a potentially higher rate of returning to sports, although differences in re-rupture rates and sural nerve injuries were not significant.
Attia et al. (19)	– MIS had a statistically significant lower rate of superficial infections (RR = 5.70, *P* < 0.001) – Faster surgical time in MIS group (29.7 vs. 51 min)	– Non-significant difference in total complication rate between groups (RR = 1.50, *P* = 0.14) – Sural nerve palsy only reported in MIS group (3.4%, *P* = 0.02) – Comparable re-rupture rate between groups (*P* = 0.50).	MIS associated with shorter surgical times and lower superficial infection rates but higher temporary sural nerve palsy. Functional outcomes and re-rupture rates largely similar.
Del Buono et al. (20)	– Higher range of motion in patients undergoing percutaneous repair (significantly greater than open repair) – Lower complication rates observed in minimally invasive surgery compared to open surgery. – Cost analysis favored minimally invasive surgery, being more economical (£558 vs. £1681 per procedure). – Better cosmetic outcomes reported in minimally invasive surgery with smaller incisions (3.4 cm vs. 12 cm) and higher patient satisfaction regarding scar appearance.	– Nerve injuries were marginally more frequent in the minimally invasive group.	Minimally invasive surgery demonstrates a better range of motion, fewer complications, and more cost-effective solutions with superior cosmetic outcomes, despite a slightly increased risk of nerve injuries compared to open surgery.
Deng et al. (21)	– Both surgical methods significantly reduced rerupture rates compared to conservative treatment (RR 0.27 for open repair and 0.14 for minimally invasive surgery).	– No significant difference in rerupture rates between minimally invasive surgery and open repair (RR 0.72, 95% CI 0.10–4.4) – Higher infection rates noted in open repair compared to conservative treatment – Significant difference in DVT incidents, favoring conservative treatment over open repair – No significant differences in sural nerve injury rates across treatments.	Both minimally invasive and open repair effectively reduce rerupture rates compared to conservative management, with no significant difference between the two surgical techniques. However, minimally invasive surgery might offer a slight advantage in reducing reruptures and complications compared to open repair.
Gatz et al. (22)	– MIS: shorter surgeries, reduced wound necrosis (OR = 3.01), fewer adhesions (OR = 4.10), and lower infection rates (ORs = 3.90, 2.01)	– Open repair: lower palpable knots (OR = 0.10), and sural nerve palsy (OR = 0.45) – No difference in re-rupture rates (OR = 1.10)	MIS offers quicker surgeries with fewer complications; open repair reduces palpable knots and nerve palsy; both methods equally prevent re-ruptures.
Grassi et al. (23)	Lower risk of complications (RR = 0.21), lower wound infection risk (RR = 0.15)	No significant difference in rerupture or other listed outcomes.	Minimally invasive surgery is associated with fewer complications and better subjective outcomes but has high study heterogeneity and risk of bias.
McMahon et al. (24)	Fewer superficial infections in MIS, better subjective outcomes in MIS	No significant difference in re-ruptures or deep infections	MIS may result in fewer superficial infections and improved subjective outcomes with no significant difference in other serious complications compared to open surgery.

Clear procedural benefits of MIS were demonstrated by Attia et al. (19) who observed that MIS had a much reduced rate of superficial infections (RR = 5.70, *P* < 0.001) and a shorter surgical time (29.7 vs. 51 min). Sural nerve palsy was only reported in the MIS group (3.4%, *P* = 0.02), and the overall complication rate did not significantly differ between the groups (RR = 1.50, *P* = 0.14). As the re-rupture rate did not differ between the groups (*P* = 0.50), there was no durability detriment.

In terms of range of motion, complication rates, cost-effectiveness (£558 vs. £1681 per treatment), and cosmetic outcomes (incision size 3.4 cm vs. 12 cm and improved scar appearance), Del Buono et al. (20) found that MIS produced better results. On the other hand, nerve injuries were slightly more common in the MIS group, indicating a possible cause for concern.

When compared to conservative treatment, Deng et al. (21) found that both surgical techniques considerably decreased the rates of re-rupture. On the other hand, re-rupture rates did not significantly differ between open repair and MIS (RR = 0.72, 95% CI 0.10–4.4), suggesting equal efficacy. The study also found no discernible variations in the rates of sural nerve injury among therapies, but greater infection rates in open repair when compared to conservative treatment.

The procedural advantages of MIS were highlighted by Gatz et al.'s (22) finding that it was linked to shorter procedures, less wound necrosis, less adhesions, and decreased infection rates. On the other hand, open repair led to less palpable knots and sural nerve palsy, indicating that there may be some advantages over MIS in these particular outcomes. Re-rupture rates did not differ (OR = 1.10), confirming comparable efficacy in this crucial parameter.

Grassi et al. (23) and McMahon et al. (24), who found improved subjective outcomes and decreased risks of complications and wound infections, further bolstered the benefits of MIS. Nonetheless, these investigations did not find any appreciable variations between MIS and OS in terms of re-ruptures or deep infections.

Functional recovery endpoints were prespecified and extracted as validated patient-reported measures (e.g., disease-specific functional scores) and performance metrics; these were analyzed alongside complications and operative time to provide a comprehensive comparison between minimally invasive surgery (MIS) and open surgery (OS). Across time points where quantitative synthesis was feasible, differences between MIS and OS in functional recovery were not consistent and, when present, were small and below commonly accepted thresholds for clinical importance; qualitative synthesis suggested a possible early advantage for MIS that diminished by later follow-up, yielding overall functional equivalence between approaches.

## Discussion

The majority of included reviews (18–20, 22–24) largely corroborates the advantages of MIS with regard to lower rates of complications, quicker recovery periods following surgery, and improved subjective and aesthetic results. On the other hand, the closeness in re-rupture rates amongst studies (18, 19, 21, 22) highlights an important area where MIS and OS functioned similarly. The modest difference in the incidence of nerve damage (19, 20) draws attention to a subtle drawback of MIS. Therefore, even while the general conclusion supports MIS due to its lower complication profile and effectiveness, the surgical team's specific experience and the characteristics of each patient should still be taken into account when selecting a surgical approach.

Significant benefits of MIS were noted by Alcelik et al. (18) and Attia et al. (19) in terms of lower surgery times and complication rates, respectively. Del Buono et al. (20), who also reported fewer problems and other benefits including cost-effectiveness and better cosmetic outcomes with MIS, support these findings. Similarly, MIS was linked to shorter surgical times and fewer sequelae, especially wound necrosis and infections, according to Gatz et al. (22). All of these research point to the conclusion that MIS is typically less complicated than OS.

Alcelik et al. (18), Attia et al. (19), and Deng et al. (21) revealed no significant differences in re-rupture rates between MIS and OS in terms of functional results. This result was corroborated by Gatz et al. (22), who showed a consistent pattern in these investigations about the similarity of both surgical approaches in preventing re-ruptures of the Achilles tendon. This is an important point since it implies that both methods retain the structural integrity of the tendon repair to an equivalent degree.

While Del Buono et al. (20) did identify a marginally elevated risk of nerve injury with MIS, Attia et al. (19) specifically noted a greater prevalence of transitory sural nerve palsy with MIS, a conclusion that was not substantially supported by other included reviews. This points to a possible area where MIS and OS could diverge adversely, even if the impact seems to be quite small and cohort-specific.

MIS use was bolstered by broader support from Grassi et al. (23) and McMahon et al. (24), who noted improved subjective outcomes and fewer problems. Though the data generally support the benefits of MIS, Grassi et al.'s (23) discussion of study heterogeneity and bias introduces a caveat, indicating that results should be interpreted cautiously even though the quality of the evidence may vary.

“MIS” represents a family of techniques—including percutaneous, mini-open, and endoscope-assisted approaches—that differ in access, visualization, and soft-tissue handling, and thus in complication profiles and convalescence trajectories. Mini-open repair, which preserves direct visualization of the Achilles tendon, has been reported to mitigate sural nerve injury relative to purely percutaneous strategies (4–7). Consequently, the choice between mini-invasive and open repair should be individualized to patient anatomy, activity demands, and surgeon expertise, recognizing that technique-specific trade-offs—rather than a simple binary hierarchy—are likely to govern outcomes (23).

MIS was observed to have had a significantly decreased rate of wound necrosis and tissue infections. This is noteworthy because tendon re-rupture is a typical source of these problems, which can lead to revision surgeries. Grassi et al. (23) found that moving from an open surgical approach to a minimally invasive one could avoid one infection out of every ten procedures. The benefits of MIS in lowering infection rates, however, might only apply to superficial infections rather than deep ones, according to conflicting results by Yang et al. (25).

It is recommended that the wound area of ATR procedures be minimised in clinical settings. Poor skin perfusion usually occurs over the Achilles tendon, which raises the risk of wound necrosis and subsequent superficial infections (26). Furthermore, these risks can be increased by specific risk factors including diabetes, vascular disease, or smoking (27). On the other hand, there has been no discernible decrease in infection rates with the administration of perioperative prophylactic antibiotics (1, 28).

One further benefit of MIS is that it operates faster. The average time for both MIS and open operations was less than 60 min, according to an analysis of three trials that included pertinent data. This suggests that the use of tourniquets or general anaesthesia had little effect on the surgical outcome. Economically speaking, the shortened surgical duration is advantageous as well, demonstrating the necessity for cost-effectiveness. In contrast, even subtracting the theatre visit, the total expenses related to open tendon restoration are almost twice as high as those for minimally invasive techniques (29).

Our conclusions about the benefits of MIS are corroborated by Patel et al. (30), who report better functional results and less soft tissue problems with MIS than with OS. In line with our findings that nerve damage is more common in MIS, this study further emphasises the possibility of sural nerve injury as a side effect of percutaneous procedures. They also highlight the functional advantages of MIS, which supports our findings about the effectiveness and security of MIS against OS.

By contrasting operational and nonoperative therapy, Ochen et al. (31) offer a more comprehensive viewpoint. Their meta-analysis revealed—a crucial finding that our evaluation did not specifically address—that operational treatments considerably lower the probability of re-rupture as compared to nonoperative methods. They also reported a greater rate of complications following operational therapies, mostly from infections, which is in line with our finding that OS had higher rates of complications than MIS. By adding comparisons of nonoperative treatments and highlighting the advantages of surgical therapies even with the higher risk of specific sequelae, this study helpfully broadens the context of our findings.

Consistent with our findings, Seow et al. (32) examined re-rupture rates across several surgical techniques and discovered no discernible variation between OS and MIS or percutaneous repair. They did point out, nevertheless, that conservative treatment usually had reduced rates of complications, with the exception of re-ruptures, which presents an intriguing contrast to our concentration on surgical procedures alone. The decision-making process for Achilles tendon rupture therapies is further complicated by Seow et al.'s (32) emphasis on the lower complication rates of conservative treatments. This suggests that therapy selection may be altered by placing a higher priority on fewer overall complication risks.

### Limitations

The limitations of this umbrella review are varied and demand careful study to contextualize the findings within an acceptable framework of scientific investigation. First and foremost, a major obstacle was the intrinsic heterogeneity across the included meta-analyses and systematic reviews. The precision of the comparative effectiveness MIS and OS may have been weakened due to differences in study design, populations, and outcome measures among the primary studies included in these reviews. Furthermore, even though the evaluation contained research from several international contexts, the results may not be universally applicable because to differences in surgical experience, hospital environments, and patient demographics. Surgical outcomes are frequently strongly correlated with the experience of the surgeon and the particular techniques used, which might range greatly in various geographical and clinical contexts. Furthermore, this review did not perform a de-novo meta-analysis. The umbrella design, overlap of primary randomized trials across included reviews, and variability in comparator definitions and outcome measures precluded valid quantitative pooling; findings were therefore integrated using a structured qualitative synthesis aligned with predefined outcomes.

### Clinical recommendations

It can be advised that MIS be taken into consideration as the preferred surgical approach for conditions where it is applicable, based on our findings. The research indicates that MIS can provide cost-effectiveness and improved cosmetic outcomes in addition to decreasing surgery times and postoperative problems such wound necrosis and infections. It's crucial to remember, nevertheless, that these benefits shouldn't be the only factors considered while deciding between MIS and OS. The comparable rates of re-rupture observed in MIS and OS cases suggest that both strategies function similarly in preserving the structural integrity of surgically repaired tissue, including Achilles tendon ruptures.

This equivalency emphasises that, rather than the intrinsic superiority of one approach over another, the choice between MIS and OS can frequently depend more on the unique patient characteristics and the surgical team's experience with each method. Furthermore, even though MIS typically has a positive profile, it is important to keep in mind that MIS is associated with a slightly higher incidence of nerve injury, such as sural nerve palsy. This implies that while choosing MIS, surgeons should carefully examine the danger of nerve damage, particularly in procedures where there is a high risk of nerve damage.

Future work should include adequately powered head-to-head randomized trials with standardized, validated functional endpoints and predefined minimal clinically important differences; uniform definitions for complications and adverse events; longer follow-up to capture durability and late sequelae; economic evaluations comparing resource use between MIS and OS; and, where feasible, individual participant data meta-analyses to explore effect modification by patient, lesion, and surgeon factors.

## Conclusion

As per our analysis, when compared to OS, MIS for acute ATR usually entails fewer problems and shorter recovery periods without sacrificing the ability to avoid tendon re-ruptures. MIS is linked to a marginally increased incidence of problems connected to the nerves, most notably transient paralysis of the sural nerve. In spite of these results, the choice between MIS and OS should be customised based on the surgeon's experience and the unique circumstances of each patient. The conclusions drawn from this analysis should be regarded cautiously due to its limitations, which include a focus on short-term outcomes and diversity in study quality.

## Data Availability

The original contributions presented in the study are included in the article/Supplementary Material, further inquiries can be directed to the corresponding author.
